# Beamline Performance Simulations for the Fundamental Neutron Physics Beamline at the Spallation Neutron Source

**DOI:** 10.6028/jres.110.018

**Published:** 2005-06-01

**Authors:** P. R. Huffman, G. L. Greene, R. R. Allen, V. Cianciolo, R. R. Huerto, P. Koehler, D. Desai, R. Mahurin, A. Yue, G. R. Palmquist, W. M. Snow

**Affiliations:** North Carolina State University, Raleigh, NC; Oak Ridge National Laboratory, Oak Ridge, TN; Oak Ridge National Laboratory, Oak Ridge, TN; The University of Tennessee, Knoxville, TN; Oak Ridge National Laboratory, Oak Ridge, TN; The University of Tennessee, Knoxville, TN; North Carolina State University, Raleigh, NC; Indiana University, Bloomington, IN

**Keywords:** McStas, neutron optics, neutron sources

## Abstract

Monte Carlo simulations are being performed to design and characterize the neutron optics components for the two fundamental neutron physics beamlines at the Spallation Neutron Source. Optimization of the cold beamline includes characterization of the guides and benders, the neutron transmission through the 0.89 nm monochromator, and the expected performance of the four time-of-flight choppers. The locations and opening angles of the choppers have been studied using a simple spreadsheet-based analysis that was developed for other SNS chopper instruments. The spreadsheet parameters are then optimized using Monte Carlo techniques to obtain the results presented in this paper. Optimization of the 0.89 nm beamline includes characterizing the double crystal monochromator and the downstream guides. The simulations continue to be refined as components are ordered and their exact size and performance specifications are determined.

## 1. Introduction

**Fig. 1:**
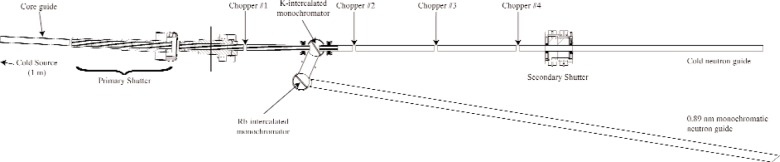
The fundamental neutron physics beamline facility. Neutrons emerge from the cold source located approximately 1 m to the left of the core guide. The length of the cold guide is approximately 14 m. The 0.89 nm monochromatic beam guide extends to a distance of approximately 40 m from the face of the cold source.

The fundamental neutron physics beamline (FnPB) is presently being constructed on cold-neutron flight path number 13 (FP13) at the Spallation Neutron Source (SNS) in Oak Ridge, TN, USA. There will be two neutron guides; one guide will terminate at approximately 15 m inside the SNS experimental hall, and the second guide will transmit a monochromatic 0.89 nm neutron beam to an external building located approximately 40 m away with respect to the cold source. The cold beam will be used for measurements that require a polychromatic wavelength distribution. The external facility will be used for experiments that require a highly monochromatic neutron beam at 0.89 nm to create ultracold neutrons (UCN) from single phonon scattering in superfluid ^4^He. Figure 1 gives an overview of the FnPB and its components.

Monte Carlo simulations are being performed in order to optimize the performance of both the cold and monochromatic beamlines. These simulations are used to specify the neutron guides (including the bending guide), the frame overlap choppers, the neutron monochromators, and other ancillary equipment. This paper outlines some of these calculations and provides some estimates of the anticipated neutron flux for the two beamlines.

## 2. Source Geometry

**Fig. 2:**
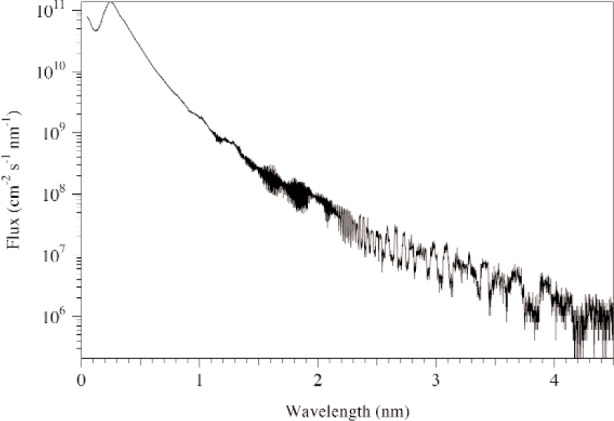
The spectrum of neutrons entering the guide. Note that at long wavelengths, there is additional noise due to the different binning procedures used in the input file and this calculation, coupled with the low statistics.

The simulations presented in this document are obtained using the neutron ray-tracing simulation package McStas [1]. The neutron source files for McStas inputs the Monte Carlo generated moderator performance estimates of the entire target system [2]. The input spectrum provided for the bottom, downstream, parahydrogen moderator is directly along flight-path number 14 and is shown in Fig. 2. Note that the spectrum for flight path 13 is not presently available but we expect minimal differences in the spectrum from the two adjacent beamlines. Calculations in this document are scaled from the input calculations performed at 2 MW to the SNS initial projected operating power of 1.4 MW.

## 3. Cold Beamline

### 3.1. Neutron Guides

The neutron guides for the cold beam are 10 cm wide by 12 cm tall, chosen to match the size of the cold moderator. The reflectivity of the guides is specified using either the measured reflectivity of the individual component, or our procurement specifications of the reflectivity given by the average reflectivity up to the Ni critical angle (*m* = 1) of 99 % and falling linearly to 65 % at *m* = 3.6. Here, *m* is the increase of the angle of total reflection, or critical angle, as compared to natural Ni.

The cold neutron guide begins 1.0 m from the face of the cold source. It consists of a 1.3 m long core guide, followed by a bender that extends approximately 5 m. The bender has five channels; there are four internal vanes running in a vertical direction dividing the horizontal dimension into five equal-sized channels. At the end of this bender, a straight *m* = 3.6 guide extends to the experimental region. Along the length of this guide there are four frame overlap choppers, a primary and secondary shutter, and a 0.89 nm monochromator.

The total length of the guide and components is 14 m, extending from approximately 1 m to 15 m from the moderator. The design is envisioned such that experiments will typically be positioned at a distance of 18 m from the moderator or 3 m from the end of the guide.

### 3.2 Neutron Choppers

To avoid frame overlap problems during the anticipated experiments, it is necessary to augment the guide with a system of choppers. For the 60 Hz operation of the SNS, the maximum wavelength range, Δ*λ*, in nm, depends on the distance, *L*, in meters, of the experiment from the source:

Δλ=6.59/L. (1)

**Fig. 3:**
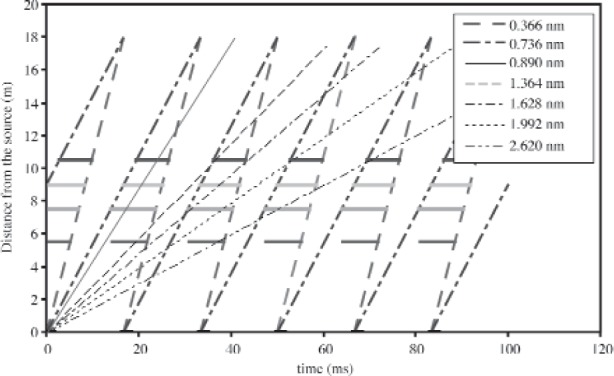
Timing diagram for chopper placement to avoid frame overlap on an 18 m long flight path determined using a spreadsheet analysis. Time is plotted horizontally and the distance from the moderator plotted vertically. The locations of the four choppers, as well as the times when they are closed, are indicated by the horizontal bars at 5.5 m, 7.5 m, 9 m, and 10.5 m. In the case shown, the choppers are phased to select the second 0.366 nm wide frame from 0.366 nm to 0.732 nm (see Fig. 4). Various diagonal lines illustrate how the choppers block slow neutrons from subsequent pulses from reaching the experiment. The line at 0.89 nm illustrates the path of the neutrons to be diffracted by the monochromator.

Our simulations indicate that in order to both eliminate slower neutrons from previous pulses from overlapping and to accommodate the extraction of the 0.89 nm neutrons after chopper #1, it is necessary to employ a system of four choppers. The locations for the four choppers are at distances of 5.5 m, 7.5 m, 9.0 m, and 10.5 m with respect to the cold source. The result of a simple optimization is shown in Fig. 3, where it is assumed that the actual experiment is placed 3 m behind the end of the guide at a total distance of 18 m from the moderator. The shortest wavelength neutrons that are in danger of not being caught by this arrangement of choppers is a band between 4.0 nm and 4.2 nm over a time band of 180 ms to 190 ms after the initial pulse. These neutrons are separated from the “good” neutrons by 10 frames and have a negligible intensity (<10^−6^) in the neutron energy spectrum.

It would be desirable to locate the monochromator crystal for the UCN beam in front of all the choppers in the cold beam so as to completely decouple the operation of the UCN beam from the operation of the first chopper on the cold beam. Unfortunately, due to space constraints associated with the FP13 beamline, the closest position available for the intercalated graphite monochromator is 6.8 m from the source, which means that the closest location for the first chopper on the cold beamline would be 7 m. This increased distance from the source makes it considerably more difficult to suppress frame overlap on the cold beam. We therefore decided that it is necessary to locate the first chopper before the monochromator. To ensure that this chopper does not interfere with full illumination of the monochromator, an extra extension of its opening time is introduced to let the 0.89 nm neutrons through the first chopper. Even with this extra open space, four choppers are still sufficient to suppress frame overlap.

**Fig. 4:**
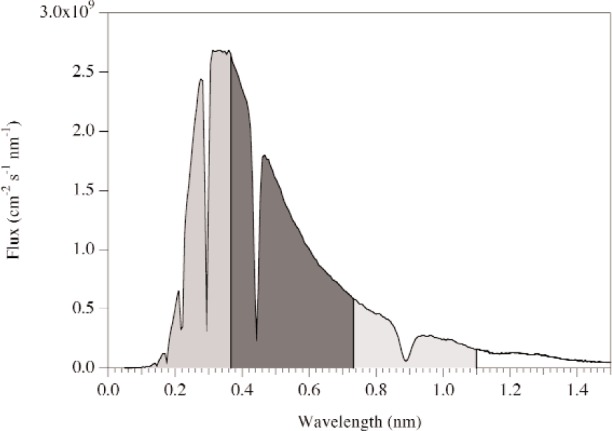
Calculated time-averaged neutron flux at the end of the proposed 10 cm by 12 cm cold guide that extends from 1 m to 15 m from the moderator. Also shown, are three wavelength regions that could be selected by choppers to avoid both frame overlap at a distance of 18 m (expected location of experiments) and the regions of fast neutrons leaking through from previous pulses (see Fig. 3). The choppers can be phased to access a more optimum region if it is determined that the background of fast neutrons from the spallation target is manageable. Note that the dips in the spectrum at 0.89 nm and higher order reflections (i.e., 0.89 nm/2) arise from presence of the upstream monochromator.

For a flight path length of 18 m, the maximum wavelength range for 60 Hz operation is 0.366 nm. To avoid potential background problems associated with the leakage of fast particles through the shielding and guide system, it may be necessary to phase the choppers so that the experiments are conducted using the wavelength regions from 0 nm to 0.366 nm, 0.366 nm to 0.732 nm, and 0.732 nm to 1.099 nm. These regions are shown graphically in Fig. 4. Note that the choppers can be timed to access a more optimum region if it is determined that the background from fast neutrons is manageable.

The spreadsheet analysis shown in Fig. 3 does not take into account the finite opening and closing times of the neutron choppers. According to the specifications for the standard SNS bandwidth-limiting neutron chopper systems, the choppers will be capable of operating at 60 Hz with 10 µs time jitter. With an outer radius of 29 cm, this chopper will take 1.5 ms to pass from one edge of the 10 cm neutron guide to the other. This time is small compared to the time-of-flight for arrival of cold neutrons at even the closest chopper and even with the addition of this opening and closing time four choppers still suffice to suppress frame overlap effects.

The opening angle for chopper #1 is set such that both 0.89 nm neutrons and an arbitrary 0.366 nm wavelength band could be accessed. This led to the rather large opening angle of 246°. The remaining three chopper angles are determined based on the three wavelength bands shown in Fig. 4. Here, the optimum opening angles are determined for each region. As expected, the angles for the three regions are similar, but not exactly the same. Thus we took the largest opening for each chopper and used this as our opening angle. The values for the opening angles for choppers #2, #3, and #4 are 150°, 180°, and 210°, respectively. Additional refinement of these parameters will be performed before final procurement of the choppers.

### 3.3 0.89 nm Monochromator

Details of the monochromator design and parameterization are discussed in detail below in Sec. 4. The transmission through the monochromator along the cold beamline is modelled using a stage-1 potassium intercalated graphite monochromator. It consists of 24 tiled intercalated graphite pieces forming a crystal that is 20 cm wide by 12 cm tall. The crystals have a mosaic of 3° and are placed at an angle such that 0.89 nm neutrons are reflected. In addition, Bragg peaks (transmission dips) occur at 0.45 nm (*λ*/2) and 0.30 nm (*λ*/3).

### 3.4 Chopper Performance

Using the opening angles for the four choppers given above, the relative and absolute phases of the four choppers were varied to obtain spectra for a number of wavelength regions. Since each experiment will use different regions and have different requirements on the amount of frame-overlap neutrons, we have chosen the region from 0.366 nm to 0.732 nm for this discussion. The performance issues we are investigating include the wavelength of the first frame-overlap neutrons, the relative fraction of these neutrons, and the timing resolution. Note that the relative fraction and timing resolution are parameters that are coupled and the phase tuning will ultimately be determined for the specific experiment at hand.

**Fig. 5:**
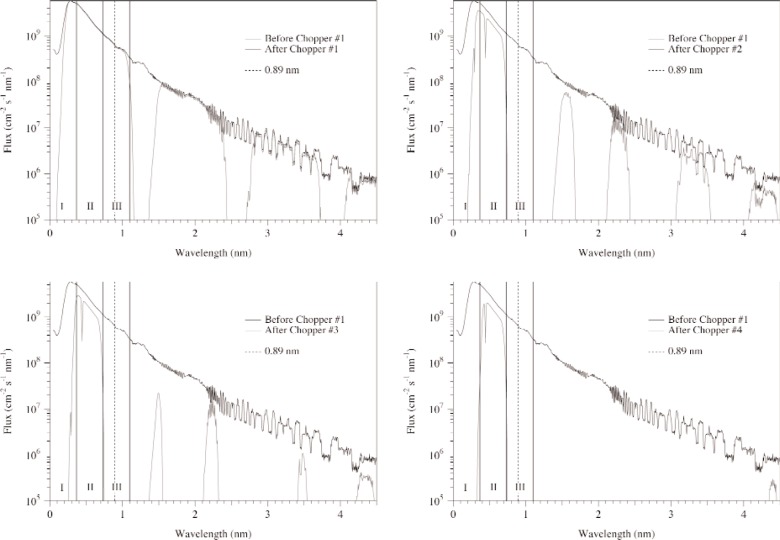
The time-averaged neutron beam spectrum after it passes through each of the four successive choppers. The regions I, II, and III correspond to the three wavelength regions shown in Fig. 4.

Figure 5 shows the effect of each of the four choppers for this region. The first frame overlap neutrons occur near a wavelength of 4.4 nm. This set of simulations was optimized to minimize frame overlap neutrons from adjacent regions at the expense of neutrons in the region of interest. The resulting phases for the four choppers are 60°, 122.5°, 170°, and 230°. The ratio of the number of neutrons in the region of interest to the total number of neutrons for this set of phase parameters is 0.9992, or the ratio of frame-overlap neutrons to neutrons in the primary pulse is 8 × 10^−4^.

## 4. Monochromatic 0.89 nm Beam for UCN Experiments

### 4.1 Overview

Between choppers #1 and #2 on the cold beam, a 0.89 nm monochromatic beam is reflected out of the beam and directed to the external facility for UCN experiments. The monochromatic beamline consists of the above-mentioned cold beamline components up to the first monochromator, continues with the second monochromator of the double-crystal design, and then with a guide to the external building. Along the last section of guide, there is a chopper to remove higher-order Bragg reflections from the crystal and a secondary shutter.

### 4.2 Monochromator Design and Optimization

The double-crystal arrangement for reflecting the 0.89 nm beam out of the cold beamline was designed based primarily on the existing National Institute of Standards and Technology (NIST) 0.89 nm monochromator [3]. There, a stage-2 potassium intercalated graphite monochromator is used to reflect out a 0.89 nm beam that is used primarily in an experiment to measure the neutron lifetime using magnetically trapped neutrons [4].

Initial benchmark simulations were performed using the existing geometry of the NIST monochromator with the input spectrum of the NIST NG-6 guide. This allowed the calculated reflectivities to be compared with measured reflectivities as a cross-check for the simulations. The calculated reflectivities agreed to within 10 % of the measured values for the reflectivity between 0.883 nm and 0.903 nm.

Different designs were then investigated to maximize the number of 0.89 nm neutrons that exit the guide in the external building. The primary complication in the design was the fact that the monochromatic beamline needs to be directed at an angle of approximately 10° with respect to the cold beamline.

The two geometries where detailed simulations were performed are: 1) a dual-crystal stage-1 potassium intercalated graphite monochromator system coupled to a downstream bender/guide system and 2) a stage-1 potassium intercalated graphite monochromator, followed by a stage-1 rubidium intercalated graphite monochromator, coupled to a straight guide. Similar performance was found between the two sets of monochromator geometries, but the latter (2) has the advantage that the downstream bender is not required, thus resulting in a greater neutron flux in addition to the substantial cost savings from not having a bender. Due to the fixed lattice-spacing for the alkali crystals, scenario 2 is the most favorable solution given the desired angle of ≈10°. Combinations using stage-1 and stage-2 crystals of potassium, rubidium, and cesium were explored. The results discussed hereafter will use geometry number 2.

The reflectivity of a single monochromator was first studied as a function of the crystal mosaic. The NIST monochromator consists of stage-2 potassium intercalated graphite crystals and is designed assuming input neutrons from a ^58^Ni (*m* = 1.2) guide, giving an optimized crystal mosaic of 1.5°.^1^ With a higher *m*-value guide, one would expect that the mosaic will need to be larger in order to more closely match the divergence of the supermirror guide. Simulations were performed using a stage-1 potassium intercalated graphite monochromator for mosaic angles of 1.5° (the approximate mosaic of the NIST monochromator), 2°, 3°, and 4°. These calculations assume intrinsic reflectivities of 85 % for both the K and Rb crystals [5]. The neutron reflectivities for the system for neutrons with wavelengths between 0.883 nm and 0.903 nm are 40 %, 52 %, 64 %, and 70 % for the respective mosaic angle. The reflectivity for a mosaic of 3° (which is roughly matched to the divergence of the *m* = 3.5 guide at 0.89 nm, 3.1°) provides the maximum fluence at the target area and is in a reasonable range for the mosaic value in terms of production.

The Bragg angle for the stage-1 K monochromator is 56.6°. Thus the reflected beam is directed at an angle of 113° with respect to the incident beam. The second monochromator (stage-1 Rb) is placed at a distance of 68 cm (center-to-center) from the first monochromator. This geometry minimizes the extra divergence arising from the monochromators themselves. Placement of a neutron guide between the monochromators does little to increase the transmission, and has the disadvantage that it increases the divergence of the single monochromator. The increased divergence arises from the fact that after the first crystal, the beam has the divergence of both the neutron guide and the monochromator. The geometry of the second monochromator cancels to first order the divergence of the first crystal, leaving an outgoing beam having a divergence equal to that of the entrance guide. When a neutron guide is placed between the two monochromators, it removes the cancellation of the divergence. Thus, with a guide, the divergence coming off the second monochromator is larger than the scenario when the guide is not present.

**Fig. 6:**
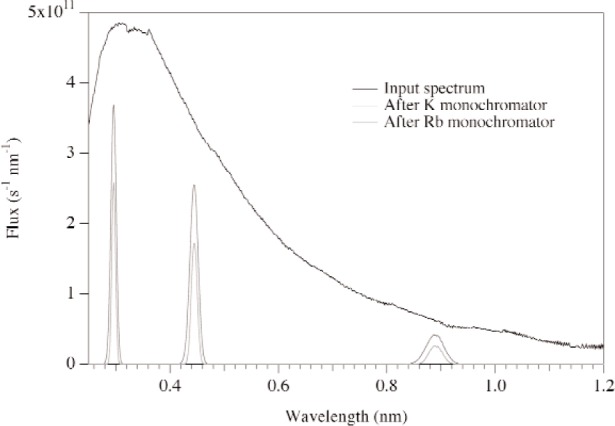
Time-averaged spectra of neutrons reflected from the both the first monochromator and the double monochromator configuration.

The simulations of the two-monochromator system are shown in Fig. 6. The curves correspond to the input spectrum, the spectrum after the first monochromator, and the spectrum after the second. These simulations are for crystals with a mosaic of 3°. The total reflectivity of this double-crystal arrangement is approximately 39 %.

In principle, one can vary the angles (or curvature) of the individual crystals to focus the beam, thereby increasing the number of neutrons incident on the final apparatus. This technique is used in many similar geometries for condensed matter instruments. Optimization of the focusing angles for the crystals is planned, but detailed simulations have not been performed at the present time. Initial estimates using a very simple geometry indicate that the flux could be increased by as much as 10 % to 20 % using this technique.

### 4.3 Downstream Guides

The 0.89 nm neutron beam emerging from the second monochromator is coupled into an ≈33 m long downstream guide to transport the beam to the external facility. The direction of this guide with respect to the upstream guide (before the first monochromator) is rotated by approximately 10° so that the guide extends along the exterior shielding wall. As described above, by using a double monochromator with different lattice or *d*-spacings, the beam can be transported without the use of a bender, thereby increasing the flux.

**Fig. 7:**
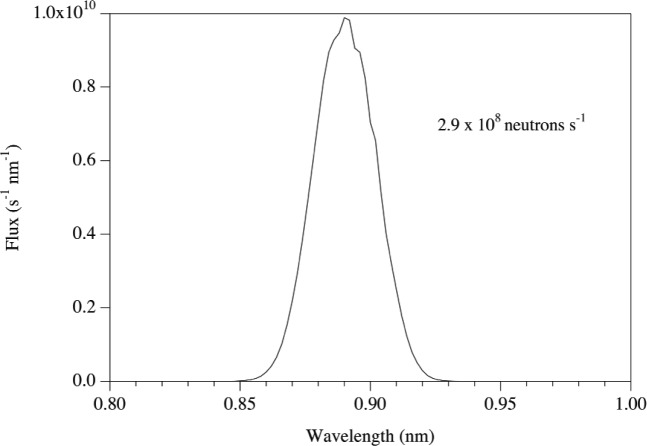
Time-averaged spectra of neutrons emerging from the 14 cm by 16 cm guide in the external building.

The present simulations use a straight *m* = 3.6 downstream guide that is 14 cm wide by 16 cm tall, chosen to closely match the size of the beam exiting the second monochromator. The neutrons undergo a significant number of reflections down this guide that, coupled to the finite reflectivity of the guide, causes a significant attenuation of the beam. Only ≈40 % of the neutrons entering the guide traverse this distance. Thus, with the present geometry, approximately 85 % of the neutrons are lost before reaching the external facility. A plot of the 0.89 nm beam is shown in Fig. 7.

Because of the long length of the downstream guide, a ballistic guide [6] could in principle be used in place of the *m* = 3.6 guide. The ballistic guide would consist of three sections, the up-taper, the center section, and the down-taper. The function of the up-taper is to make the paths of the neutrons reflecting from its walls more nearly parallel to the overall guide direction. Hence, these neutrons will strike the center section of the guide at shallower angles and also make fewer collisions in the central section. As a result, the losses in the central section can be reduced compared to a non-ballistic guide, even if this section is made of Ni rather than a supermirror. Initial simulations indicate that gains of up to 50 % in the 0.89 nm transmission could be obtained from a ballistic guide geometry. In addition, this geometry could be well-suited for the neutron EDM experiment [7].

## 5. Conclusions

The above simulations are part of an on-going project to design, specify, and procure neutron optics components for the fundamental neutron physics beamline. The performance baselines set for the peak of the cold beam and for the 0.89 nm monochromatic beam are given in Table 1. At present, the simulations show that we should be well above these baselines provided that the calculated input spectrum is reliable.

## Figures and Tables

**Table 1:** The performance baselines and simulated neutronics performance for the two fundamental neutron physics beamlines

	*ϕ*_cold_ (cm^−2^s^−1^ nm^−1^ MW^−1^)	*ϕ*_0.89_ (s^−1^ nm^−1^MW^−1^)
Performance baselines	4.2 × 10^6^	2.1 × 10^7^
Simulated performance	1.9 × 10^7^ (at 0.34 nm)	2.6 × 10^8^

## Footnotes

1 The mosaic structure of the crystal is the substructure into which a “single” crystal is broken up into a number of tiny blocks, each slightly disoriented with respect to each other. The mosaic angle relates to the gaussian distribution of the normal planes of the tiny crystals.
